# Flat nonlinear optics with intersubband polaritonic metasurfaces

**DOI:** 10.1515/nanoph-2024-0742

**Published:** 2025-03-21

**Authors:** Jonas H. Krakofsky, Raktim Sarma, Igal Brener, Andrea Alù, Jongwon Lee, Mikhail A. Belkin

**Affiliations:** 9184 Walter Schottky Institute, Technical University of Munich, Garching 85748, Germany; Center for Integrated Nanotechnologies, Sandia National Laboratories, Albuquerque, NM 87123, USA; Photonics Initiative, Advanced Science Research Center, City University of New York, New York, NY 10031, USA; Physics Program, Graduate Center, City University of New York, New York, NY 10016, USA; Department of Electrical Engineering, Ulsan National Institute of Science and Technology (UNIST), Ulsan 44919, Republic of Korea

**Keywords:** second harmonic, optical power limiting, flat optics, metasurfaces, intersubband nonlinearities, gradient metasurface

## Abstract

Nonlinear intersubband polaritonic metasurfaces produce some of the strongest second- and third-order nonlinear optical responses reported for condensed matter systems at infrared frequencies. These metasurfaces are fabricated as two-dimensional arrays of nanoresonators from multi-quantum-well semiconductor heterostructures, designed to produce strong nonlinear responses associated with intersubband transitions. By optimally coupling the optical modes of the nanoresonators to vertically polarized intersubband transitions in semiconductor heterostructures, one can boost the nonlinear response associated with intersubband transitions, make intersubband transitions interact with free-space radiation at normal incidence, and hence produce optically thin flat nonlinear optical elements compatible with free-space optical setups. As a result of the strong nonlinear response in these systems, significant nonlinear conversion efficiencies (>0.1 %) can be attained in deeply subwavelength optical films using modest pumping intensities of only 10–100 kW/cm^2^. Subwavelength metasurface thickness relaxes phase-matching constraints limiting the operation of bulk nonlinear crystals. Furthermore, the amplitude and phase of the nonlinear optical response in intersubband polaritonic metasurfaces can be tailored for a specific pump wavelength and a nonlinear process of interest through the co-optimization of quantum engineering of electron states in semiconductor heterostructures and photonic engineering of the metasurface nanoresonators design. Additionally, an applied voltage can dynamically control the amplitude and phase of the nonlinear optical response at a nanoresonator level. Here, we review the current state of the art in this rapidly expanding field, focusing on nonlinear processes supporting second-harmonic generation, saturable absorption, and optical power limiting.

## 1 Introduction

Not one but two burgeoning fields of modern optics – intersubband optoelectronics and metasurface optics – owe their development from fundamental concepts to practical applications to the pioneering work of Prof. Federico Capasso, whose birthday we celebrate in this special issue of Nanophotonics. The terms “quantum cascade lasers” (QCLs) and metasurface-based “flat optics,” coined in Prof. Federico Capasso’s laboratories, are now broadly accepted terms underpinning these two exciting fields of science and technology.

**Figure 1:**
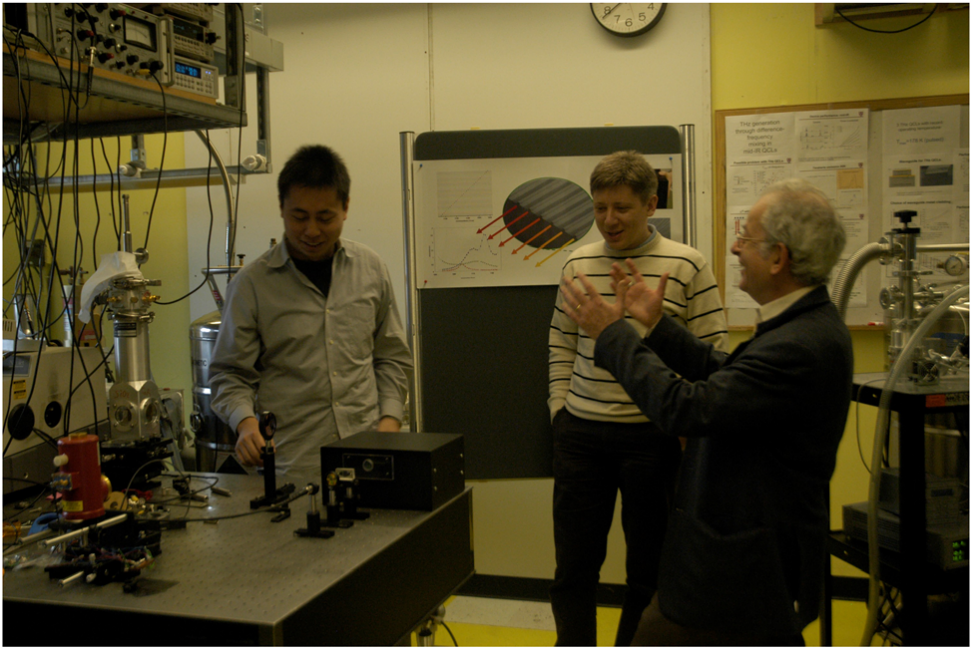
An image from a 2007 article from the student-run newspaper The Harvard Crimson [4]. The original image title reads: “Professor Federico Capasso praises his Quantum Cascade Laser as research associate Mikhail Belkin and graduate student Benjamin Lee look on.” Reproduced with permission from The Harvard Crimson.

One of the coauthors of this review article (MAB) had the honor of joining Prof. Capasso’s group 1 year after he transitioned from Bell Labs to Harvard. Back then, Prof. Capasso was best known as the inventor of QCLs [1], Figure 1. The concept of metasurface-based flat optics was not yet developed [2], [3] at that time. Another coauthor (IB) worked in the same division as Prof. Capasso at Bell Labs in Murray Hill for nearly a decade. IB was very close to Capasso’s group, and a close friend of several generations of his successful postdocs. Through that period, Capasso was legendary for his work ethics and enthusiasm for bandgap engineering.

The field of intersubband optoelectronics and the field of metasurface optics have little in common in terms of materials, design approaches, and fabrication methods, while being fundamentally connected by the general concept of using a top-down design-based approach to tailoring electrical and optical properties of materials through nanostructuring.

This paper overviews the state of the art in the field of nonlinear intersubband polaritonic metasurfaces (IPM). The development of IPMs was inspired by the success of flat optics and the knowledge developed by the field of intersubband optoelectronics [5]. Nonlinear IPMs bring together the fields of intersubband optoelectronics and metasurface optics by combining quantum engineering of electronic states in semiconductor heterostructures (as in QCL design) and electromagnetic engineering of optical nanocavities on flat substrates (as in flat optics), to produce metasurfaces with a nonlinear response that can be many orders of magnitude larger than that of traditional nonlinear optical materials. The giant nonlinear response achieved in IPM enables nonlinear wave mixing in deeply subwavelength films with relaxed phase-matching constraints and with control over the phase and amplitude of the nonlinear response at the individual nanoresonator level.

## 2 Intersubband polaritonic metasurfaces for wave mixing

The resonant transitions between electron states confined in a multiple quantum well (MQW) heterostructure are known as intersubband transitions. Due to the relatively small effective masses of electrons in many semiconductor well materials (InGaAs, GaAs, etc.), one can achieve very large transition dipole moments (∼100 D) for transitions in the mid-infrared frequency range (mid-IR, *λ* ≈ 3–30 μm). Utilizing this property and the ability to tailor the electron wavefunctions and the transition energies in MQWs by bandstructure engineering, several reports in the late 1980s and early 1990s demonstrated MQW heterostructures that exhibit second- and third-order nonlinear susceptibilities 3 to 5 orders of magnitude higher than those of natural nonlinear crystals [6], [7], [8], [9], [10].

The transitions between electron subbands in the MQW heterostructures support dipole moments polarized mainly in the surface-normal direction (growth direction), and the strongest elements of the nonlinear susceptibility tensor associated with intersubband transitions are also along the same direction. Assuming this is directed along the *z* coordinate axis, the nonlinear susceptibility tensor components $\chi_{\mathrm{zzz}}^{\left(2\right)}$ or $\chi_{\mathrm{zzzz}}^{\left(3\right)}$ are by far the largest.

By contrast, the intersubband nonlinear response of MQWs nearly vanishes for light incidence normal to the wafer surface. As a result, following the initial demonstration of the giant nonlinearity in MQWs using free-space optical setups, further research mostly focused on exploiting intersubband nonlinearities in waveguide geometries, often integrated with QCL active regions, e.g., for second-harmonic generation (SHG) [11] or THz difference frequency generation (DFG) [12]. Applying metasurface and polaritonic coupling concepts to MQW intersubband nonlinearities enabled the development of large-area nonlinear optical elements that can be used in free-space optical setups.

A nonlinear metasurface consists of a two-dimensional array of subwavelength optical nanoresonators, known as meta-atoms, separated by subwavelength distances. Due to their subwavelength thickness, the phase-matching constraint is significantly relaxed in such devices. Moreover, the nanoresonator design can tailor the amplitude, phase, and polarization state of the nonlinear response. Thus, one can tailor the nonlinearly generated optical wavefront in a metasurface on a subwavelength scale.

One of the major challenges facing nonlinear metasurfaces is the difficulty of achieving high conversion efficiency, due to their subwavelength thickness. For efficient frequency conversion, the nonlinear polarization must be comparable to the linear response [13]. Taking as an example SHG, ${\vec{P}}^{\left(2\right)}=\varepsilon_{0}{\overset{↔}{\chi }}^{\left(2\right)}{\vec{E}}_{\omega }{\vec{E}}_{\omega }$ , this requirement translates into the need for the product of the effective second-order nonlinear response $\chi_{\text{eff}}^{\left(2\right)}$ , determined for a given input/output polarization combination, and the pump *E*-field intensity *E*_pump_ to be close to 1, $\left|\chi_{\text{eff}}^{\left(2\right)}E_{\text{pump}}\right|\approx 1$ [13].

**Figure 2:**
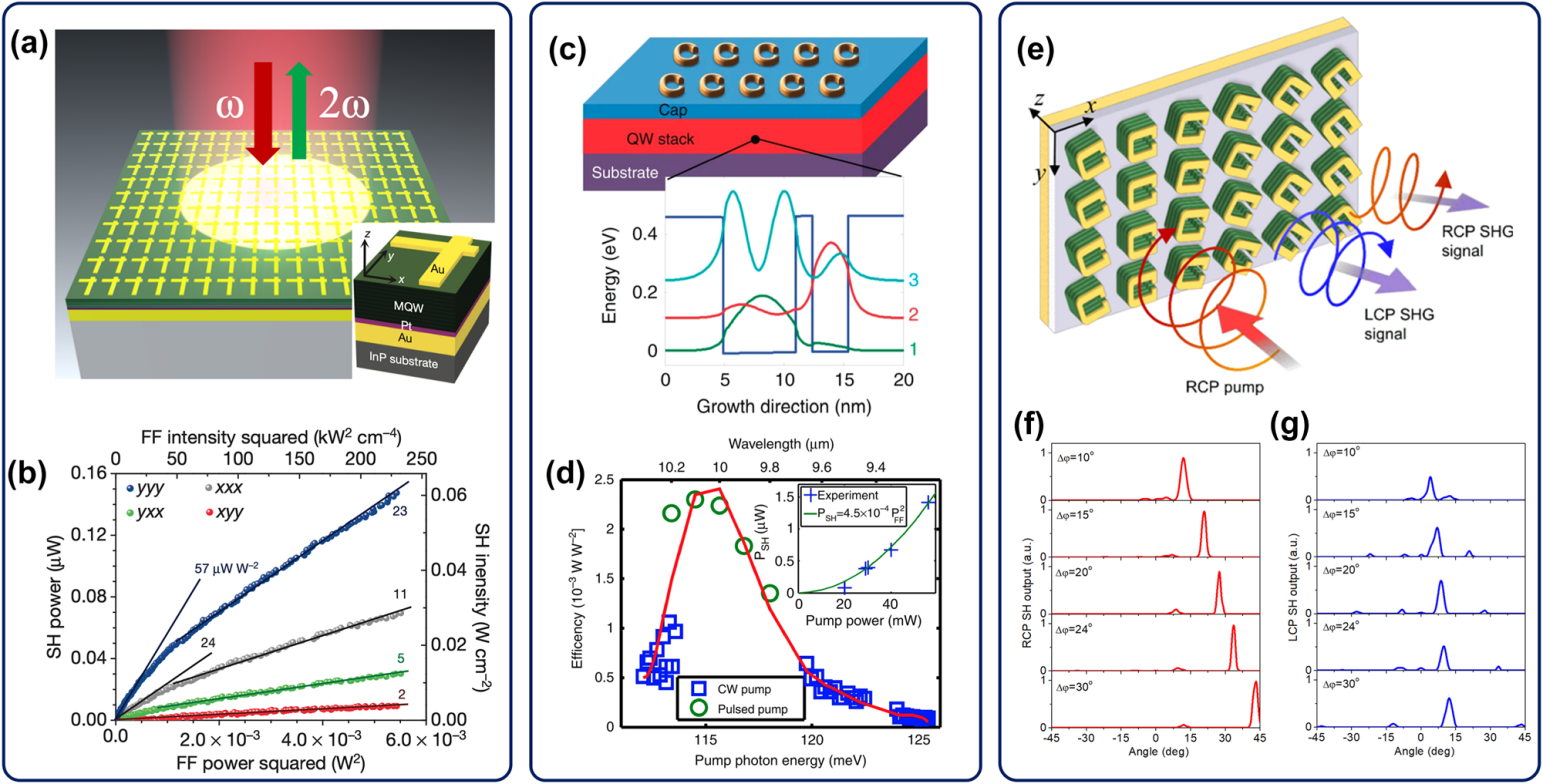
IPMs for SHG based on metal nanoresonators coupled to MQW heterostructures. (a, b) Schematic of an IPM for SHG using plasmonic Γ-shaped nanocavity array (Inset: meta-atom unit structure) (a) and experimentally measured second-harmonic signal from the metasurface (b) [5]. (c, d) Schematic of an IPM for SHG using plasmonic split-ring-resonator array (Zoom-in view: conduction band edge and computed electron subbands envelope wavefunctions of a single period of the MQW heterostructure) (c) and experimentally measured SHG conversion efficiency of the metasurface (d) [14]. (e) Schematic of PB IPM for local phase control of the SHG signal [15]. (f, g) Experimental far-field profiles of RCP (f) and LCP (g) second-harmonic output from PB phase IPM with different phase gradient values [16]. Panels (a, b) are reproduced with permission from Ref. [5]. Copyright 2014, Springer Nature. Panels (c, d) are reproduced with permission from Ref. [14]. Copyright 2015, Springer Nature. Panel (e) is reproduced with permission from Ref. [15]. Copyright 2014, American Physical Society. Panels (f, g) are reproduced with permission from Ref. [16]. Copyright 2016, Optica.

The intrinsically low nonlinear optical response of traditional nonlinear materials requires pump field intensities above the material damage threshold to satisfy this condition (unless ultra-short laser pulses are used). Nonlinear IPMs solve this problem by offering a nonlinear response many orders of magnitude larger than the one of traditional nonlinear crystals. In the first demonstration [5], a metasurface was designed by placing a 400 nm-thick In_0.53_Ga_0.47_As/Al_0.48_In_0.52_As MQW semiconductor heterostructure between a bottom metallic ground plane and a top Γ-shaped gold antenna in an array of meta-atom structures shown in Figure 2(a). The MQW heterostructure was designed to support a doubly resonant SHG at 8 μm pump wavelength [5], [10]. The components of the effective nonlinear susceptibility tensor of the metasurface $\chi_{\mathrm{ijk}}^{\left(2\right)e\mathrm{ff}}$ can be engineered according to the polarization response of the gold nanostructure. The values of the $\chi_{\mathrm{ijk}}^{\left(2\right)e\mathrm{ff}}$ tensor elements can be computed using the overlap integral [5]

(1) $$\chi_{\mathrm{ijk}}^{\left(2\right)e\mathrm{ff}}=\chi_{\mathrm{zzz}}^{\left(2\right)}\left[\frac{\underset{UC}{\int }\xi_{i}^{2\omega }\left(x,y,z\right)\xi_{j}^{\omega }\left(x,y,z\right)\xi_{k}^{\omega }\left(x,y,z\right)dV}{V}\right],$$

where $\xi_{i}^{\omega }$ ( $\xi_{i}^{2\omega }$ ) is the induced *z*-polarized E-field in the MQW structure normalized to the *i*-polarized incident wave at *ω* (2*ω*), $\chi_{\mathrm{zzz}}^{\left(2\right)}$ is the intersubband nonlinear response of the MQW layer, and the expression in square brackets is the nonlinear overlap integral between fundamental and second-harmonic modes of the nanoantenna with the integration going over the entire unit-cell (UC) volume [13].

These first nonlinear IPMs produced easily detectable SHG output using only mW-level optical pumping focused to produce ∼10 kW/cm^2^ intensity, see Figure 1(b). The highest second-order nonlinear response of the metasurface $\chi_{\mathrm{yyy}}^{\left(2\right)e\mathrm{ff}}\approx 55\;\text{nm}/\text{V}$ was measured for *y*-in and *y*-out SHG polarization combination, see Figure 1(a) and (b) [5]. Shortly after this demonstration, Wolf et al. also demonstrated SHG in the mid-IR range by coupling plasmonic split-ring resonators (SRR) with an MQW layer using a coupled-double-quantum-well structure shown in Figure 2(c) [14]. This work achieved SHG conversion efficiency of nearly 2.5 mW/W^2^ and reached SHG power conversion efficiency of 0.14 % using pump powers in the range of 1–10 W, see Figure 2(d) [14]. Reference [14] also showcased the ability of IPMs to create phased-array sources based on the local phase and amplitude variation of the nonlinear response produced by different meta-atoms [14]. A year later, Lee et al. demonstrated SHG in the mid-IR range using an MQW layer-loaded T-shaped plasmonic nanocavity structure [13]. By etching away the MQW regions outside of plasmonic antennas, they achieved improved near-field enhancement and modal overlap, yielding record-high values of effective nonlinear susceptibility of the nonlinear IPMs of 10^6^ pm/V at a wavelength of 10 µm and experimentally achieved conversion efficiencies up to 0.075 % with a pump powers ∼100 mW and pump intensity of only ∼15 kW/cm^2^.

We note that, while, due to experimental simplicity, most of the demonstrations of wave mixing in nonlinear IPMs focused on SHG, these metasurfaces may also be used for a wide range of other wave mixing processes. In particular, IPMs for mid-IR difference-frequency generation (DFG) were experimentally demonstrated in Ref. [17], and operation of IPMs as highly efficient nonlinear optical elements for THz DFG was theoretically discussed [18]. Furthermore, third-harmonic generation (THG) was also shown with IPMs, see, e.g., Ref. [19], and up-conversion was explored in [20].

The upper bound of the operating frequency for intersubband nonlinearity in MQW structures is determined by the conduction band offset of the heterostructure. Heterostructures produced using AlGaAs/GaAs and InGaAs/AlInAs/InP materials can only provide resonant intersubband nonlinearities in the mid-IR spectral range and at longer wavelengths. To extend the operation of nonlinear IPMs to shorter wavelengths, other material systems such as GaN/AlN, GaN/AlGaN [15], [16], [21], [22], [23], and TiN/Al_2_0_3_ [24] that offer larger conduction band offsets have been explored. Wolf et al. demonstrated nonlinear IPMs using a III-nitride MQW layer coupled with a plasmonic SRR array [25], [26]. SHG at 1.6 μm wavelength was demonstrated, and the largest tensor element of the effective second-order nonlinear susceptibility of the metasurface was measured to be 1.3 nm/V [25].

Beyond the giant nonlinearity coefficients that can be engineered in IPMs, an important metric of performance for these metasurfaces is the total generated power of the conversion product, which is fundamentally limited by saturation phenomena. By optimizing the pumping scheme together with material and photonic engineering, it is possible to limit saturation effects and enable large wave mixing not only for low input power levels but also for higher pumping, increasing the overall power levels. This approach has been explored in the context of up-conversion IPMs [20]. Larger nonlinear responses may be achieved by leveraging lattice resonances in periodic structures [27], [28].

Focusing on localized resonant responses, the amplitude and phase of the generated nonlinear output can be controlled at the meta-atom level, and one approach to local phase tuning is to use the Pancharatnam–Berry (PB) phase. This method controls the local phase response by adjusting the in-plane orientation angle of each meta-atom when using a circularly polarized (CP) input pump beam. The theoretical foundations for this method were presented by Tymchenko et al. [29], followed shortly after by the experimental demonstration of local nonlinear response phase tuning and SHG beam shaping in IPMs using the PB phase by Nookala et al. [30]. Reference [30] utilized an MQW layer-loaded plasmonic SRR cavity structure as shown in Figure 2(e). Both left- and right-circularly polarized (LCP and RCP, respectively) second-harmonic (SH) signals were generated simultaneously from an RCP input pump beam by an individual nanoresonator. Depending on the rotation angle of an individual nanoresonator of the metasurface, its LCP and RCP SH signal comes with a distinct phase. By varying the rotation angle of the nanoresonators as shown in Figure 2(e) and pumping many resonators with a normally incident pump beam, one can introduce gradient in the phase of the nonlinear optical response resulting in the steering of the SH output angle as shown in Figure 2(f) and (g).

The PB dependence of the local phase response of an RCP or LCP harmonic signal leads to selection rules imposed by the rotational symmetry of the meta-atoms composing the metasurface. More specifically, SH or third-harmonic (TH) signals generated by meta-atom structures with C3 or C4 symmetries, respectively, pumped by a circularly polarized (CP) pump beam only produce nonlinear output with the same CP as the pump beam. This was originally experimentally shown in Ref. [31] for SHG and THG in metasurfaces made of simple metal antennas. By applying this principle to nonlinear IPMs, it is possible to create nonlinear IPMs for SHG and THG in which only one CP state of the harmonic signal is generated for a given CP state of the pump beam [32]. One can then apply the PB phase concept and rotate these resonators relative to one another to control the phase of the only allowed CP SHG or THG beam [32].

If, in addition to having, C3 or C4 rotational symmetries, the nanoresonators lack in-plane mirror symmetry, the SHG and THG power output will be different for LCP and RCP pumps (the CP of the SH and TH output will stay the same as the pump CP as discussed above). By careful design of the nanoresonators, it is possible to build a SHG or THG IPMs with nearly unity circular dichroism, that is, SHG or THG output from these metasurfaces is nearly completely extinguished for a particular CP pump, while being strong for a pump beam with an opposite CP [33].

**Figure 3:**
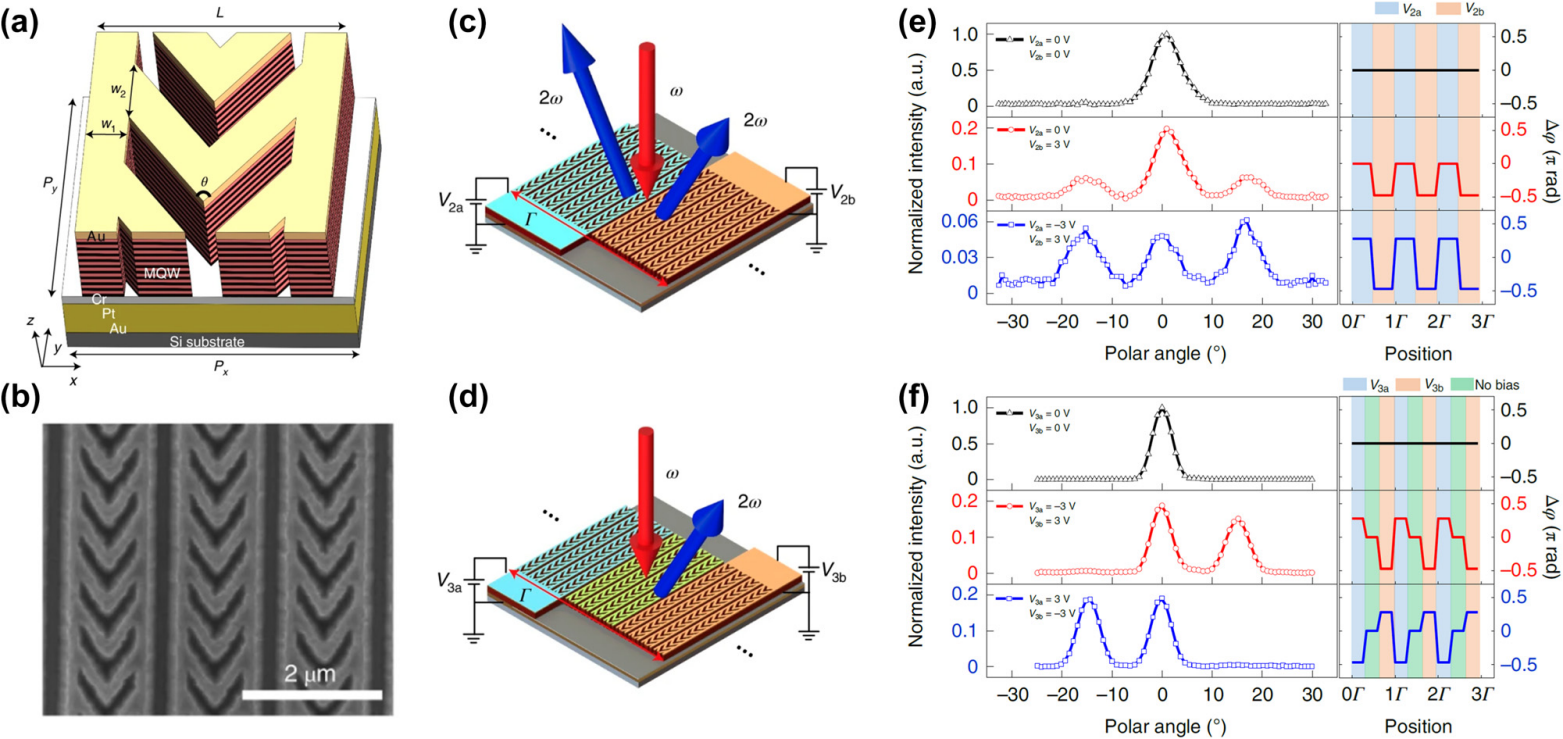
Electrically tunable IPMs for SHG. (a) Schematic of the unit cell structure consisting of a complementary V-shaped plasmonic nanocavity. (b) Scanning electron microscope image of the fabricated device. (c, d) Supercells of the metasurface consist of two (c) or three subsections (d), each one biased by a different voltage. (e, f) The far-field profiles of the SHG signals for the metasurfaces described in (c) and (d), respectively. Reproduced with permission from Ref. [34]. Copyright 2022, Springer Nature.

It is also possible to control the local nonlinear response in nonlinear IMPs by an external stimulus, such as an applied voltage. The resonant intersubband nonlinearities in MQWs can be modulated via Quantum-Confined Stark Effect (QCSE) tuning of intersubband transitions through voltage application, as was first shown by Capasso et al. in 1994 [9]. This concept was applied to nonlinear IMPs in 2022 [34] by Yu et al. Utilizing the resonant intersubband nonlinearity and QCSE among three spatially separated electron subbands in MQW structure, they demonstrated a nonlinear IMP for SHG with electrically controlled phase and amplitude of the nonlinear response. The schematic and the electron microscope image of the metasurface are shown in Figure 3(a) and (b). As shown in Figure 3(a), using a complementary V-shape antenna enabled electrical connection between many metasurface unit cells. By applying bias voltage as shown in Figure 3(c) and (d), one can introduce variation of the $\chi_{\mathrm{yxx}}^{\left(2\right)e\mathrm{ff}}$ between adjacent unit cells. The resultant voltage-induced diffraction and steering of the SH beam are shown in Figure 3(e) and (f) [34].

QCSE may also be used to create voltage-tunable IPMs for generating tunable nonlinear response for broadband SHG [35] or THG [36].

## 3 All-dielectric intersubband polaritonic metasurfaces

**Figure 4:**
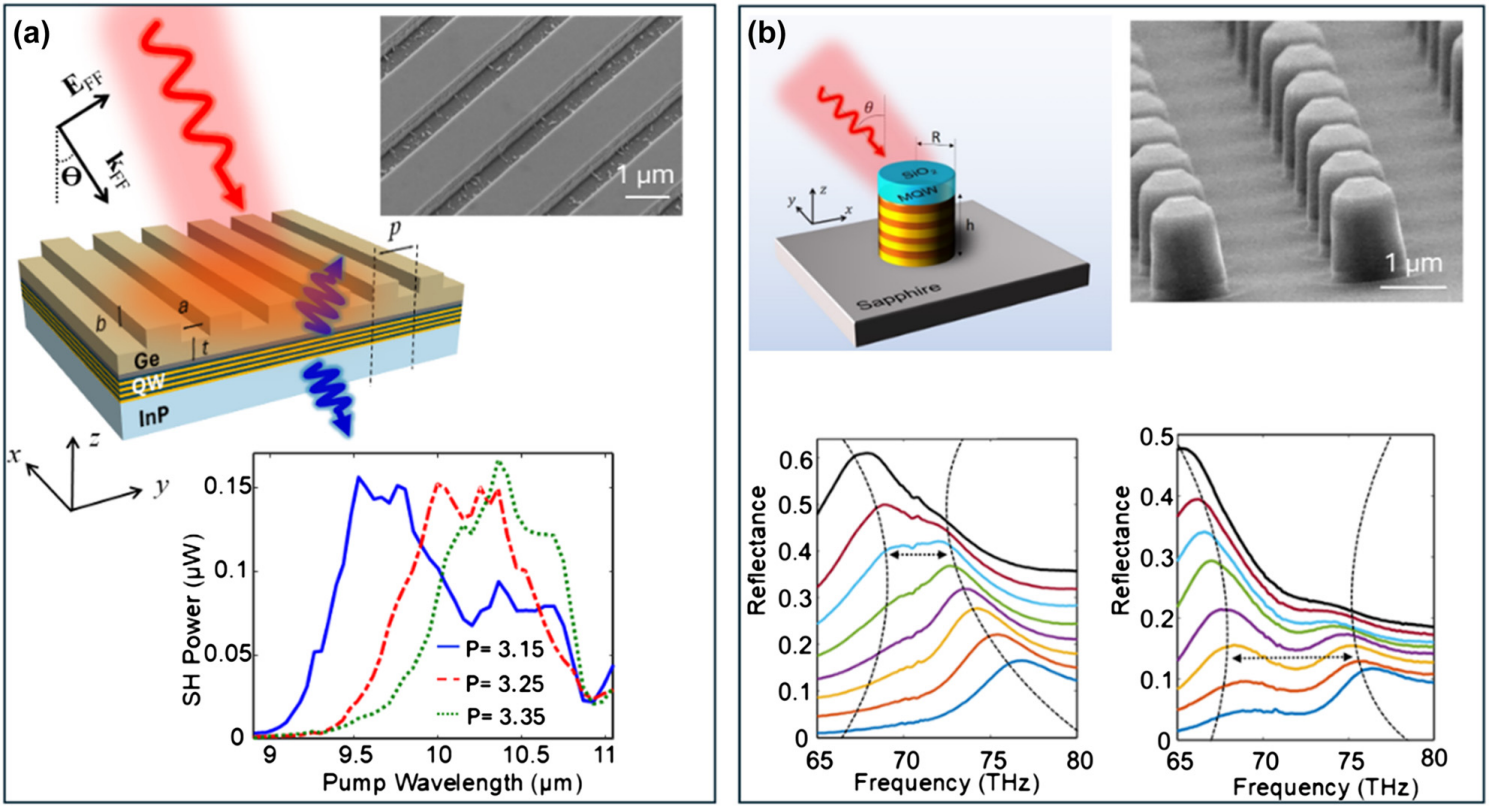
All-dielectric IPMs for SHG and strong coupling. (a) Top: Schematic and scanning electron microscope image of the hybrid all-dielectric germanium (Ge) metasurface supporting LMRs for SHG. Bottom: Experimentally measured reflected SH signal as a function of pump wavelength at normal incidence for metasurfaces with different periodicities (*p*) at a pump intensity of approximately 3.3 kW/cm^2^. Reproduced with permission from Ref. [37]. (b) Top left: Schematic of a unit cell of an all-dielectric IPM supporting Mie resonances with semiconductor heterostructure embedded within the resonator. The height of the cylindrical resonators is 1.25 µm. The radii of the cylinders are varied to spectrally tune the resonant wavelengths of the magnetic dipole Mie resonance. The figure on the top-right shows a scanning electron microscope image of one of the fabricated metasurfaces. Bottom: Experimentally measured reflectance spectra of IPMs with cylindrical resonators of different radii (vertically offset) with two different heterostructures embedded within the resonators. The dashed lines show the anticrossing behavior typical of strong coupling, with the double-sided arrows showing the values of the Rabi splitting. In all cases, the magnetic dipole Mie resonance supported by the resonator couples to the intersubband transition. Reproduced with permission from Ref. [38].

While remarkable results related to generation and control of nonlinear wave mixing were obtained using polaritonic metasurfaces comprised of metal/plasmonic nanocavities, recently there have been efforts to realize these nonlinear effects, especially second-harmonic generation, using all-dielectric metasurfaces [37], [38], [39], [40], [41]. Compared to their plasmonic counterparts, all-dielectric metasurfaces offer much lower losses and higher damage thresholds, which is crucial for nonlinear optics-based applications that often require high pump intensities. One of the first demonstrations of second-harmonic generation using all-dielectric IPMs was reported by Sarma et al. [37], which utilized high-quality-factor (high-Q) leaky mode resonances (LMRs) in germanium grating-like dielectric structures coupled to intersubband transitions in an In_0.53_Ga_0.47_As/Al_0.48_In_0.52_As MQW semiconductor heterostructure placed underneath the dielectric structure (see Figure 4(a)) [37]. The MQW heterostructure was designed for doubly resonant SHG at 10 μm pump wavelength. The dielectric structures were optimized to support LMRs at both the pump and second-harmonic wavelength for efficient second-harmonic generation and emission. The LMR resonance wavelength was spectrally tuned by either changing the period of the grating or the pump incident angle. The combination of the giant nonlinearity of the intersubband transitions and the large field enhancements associated with the high-Q LMR resonances that were slightly spectrally detuned from the intersubband resonance allowed for efficient SHG over a wide range of pump wavelengths ranging from 8.5–11 µm (Figure 4(a)). The maximum SHG conversion efficiency experimentally demonstrated using these all-dielectric intersubband polaritonic metasurfaces was about 1.1 mW/W^2^, which was orders of magnitude higher compared to SHG conversion efficiencies achieved using all-dielectric metasurfaces fabricated out of conventional semiconductors such as GaAs or AlGaAs [42], [43].

In Ref. [37], similar to plasmonic metasurfaces that enhance the fields near the surface of the resonators, the polaritonic coupling between the LMR resonance and the intersubband transitions relied on the exponentially decaying evanescent fields. Utilizing evanescent fields for coupling incident pump light to the intersubband transitions limits the ability to control the light–matter interaction volume and the spatial overlap between the photonic modes and the semiconductor heterostructure. In addition, LMRs are delocalized, making it even more difficult to efficiently control the light–matter interaction. Shortly after the demonstration of Ref. [37], in Refs. [38], [39], all-dielectric IPMs designed to support Mie-type photonic resonances were utilized to mitigate these issues. Unlike LMRs, Mie-type photonic resonances are volume resonances where a major portion of the electromagnetic fields is confined within the volume of the resonator. The resonant frequency and field distribution of the Mie resonances can be controlled using the symmetry, shape, periodicity, and size of the dielectric resonators. As a result, Mie-type photonic resonances offer a very flexible platform to control light–matter interaction for optimizing SHG efficiency [44], [45]. In these Mie-type IPMs, instead of placing the semiconductor heterostructure underneath the nanostructured dielectric layer, the heterostructure was embedded within the resonators (see Figure 4(b)). The resonators were designed to support Mie-type resonances with strong electric field components along the surface-normal direction (growth direction) to efficiently couple the normally incident pump light to the intersubband transitions.

**Figure 5:**
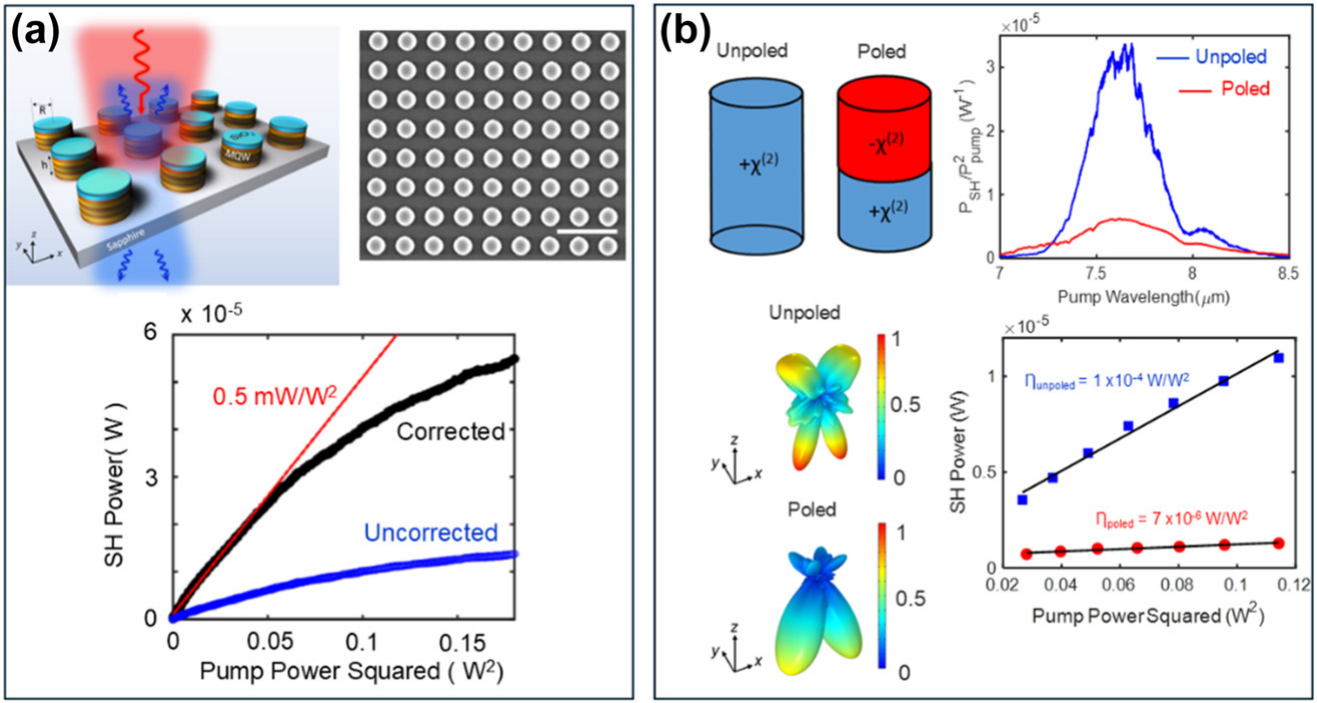
Poled and unpoled all-dielectric intersubband Mie metasurfaces for SHG. (a) Top: Schematic and scanning electron microscope image of a fabricated Mie metasurface used for SHG in Ref. [39]. The scale bar in the scanning electron microscope image is 5 µm. The metasurface comprises cylindrical resonators with the heterostructure embedded within the resonators. Bottom: Experimentally measured peak SH power as a function of the square of the pump power at a fixed pump wavelength of 7.65 µm. The figure contains raw data (uncorrected) and data corrected for the limited collection efficiency of the optical setup (corrected). The slope of the linear fit at low pump powers corresponds to the nonlinear conversion factor equal to 0.5 mW/W^2^. Reproduced with permission from Ref. [39]. (b) Top left: Schematic of the resonators of two types of metasurfaces fabricated to demonstrate the effect of polarity variation of nonlinear susceptibility. The unpoled resonator consists of a nonlinear medium with one polarity; the poled resonator consists of a nonlinear medium with two opposite polarities, each filling half the volume of the resonator. Bottom left: Calculated far-field emission intensity profile of the two types of resonators shown above. Top right: Experimentally measured reflected SH signal from the two metasurfaces plotted as a function of pump wavelength. The poled metasurface shows significantly lower SHG efficiency. Bottom right: Experimentally measured reflected SH signal from the two metasurfaces at a fixed pump wavelength of 7.65 µm plotted as a function of the square of the pump power. The nonlinear conversion factors shown by the black lines are stated in the figure. Reproduced with permission from Ref. [40].

In Ref. [38], Sarma et al. demonstrated for the first time strong coupling between Mie resonances and intersubband transitions in semiconductor heterostructures and demonstrated the ability to tailor the strength of polaritonic coupling by engineering either the semiconductor heterostructure or the Mie resonances. Finally, a unique degree of freedom that intersubband-based nonlinearity can offer is the ability to control the polarity of nonlinear susceptibility at length scales much smaller than the optical wavelength. The nonlinear susceptibility due to intersubband transitions is proportional to the product of three dipole moments, the signs of which can be flipped by reversing the sequence of the growth of the heterostructure. This implies that, by controlling the growth sequence, it is possible to control both the magnitude and the sign of the nonlinear susceptibility along the growth direction at length scales significantly smaller than the optical wavelength. Such arbitrary microscopic control is extremely hard to achieve using conventional semiconductors such as GaAs or AlGaAs that are typically used for fabricating all-dielectric nonlinear metasurfaces for SHG. This unique degree of freedom, combined with the ability to control the spatial profile of the resonances in a Mie metasurface, can offer unique abilities to control the SHG radiation pattern and conversion efficiency using nonlinear metasurfaces. This was conceptually demonstrated in Ref. [40], where two types of all-dielectric intersubband-based Mie metasurfaces were fabricated. In the first type, the resonators were made of a nonlinear material with a single polarity of the nonlinear susceptibility, and, in the second type, the resonators were made out of nonlinear material with opposite polarities (see Figure 5(b)). Because of the different spatial distributions of the nonlinear polarization within the resonators, for the two types of metasurfaces, different superpositions of modes at the second-harmonic wavelength were excited, which led to different emission patterns of SH signal and leading to an experimentally measured 5X overall difference in the SHG efficiency (Figure 5(b)).

## 4 Saturable absorbers and optical power limiters based on intersubband polaritonic metasurfaces

Nonlinear optical materials with fast and strong Kerr nonlinearity ( $\chi^{\left(3\right)}$ ) are essential for generation of ultra-short laser pulses, building optical power limiters, and constructing nonlinear optical elements for all-optical computing. Optically thin films with high Kerr nonlinearity are particularly interesting for practical applications as they allow tight light focusing on the surface. In addition to providing giant second-order nonlinear response, nonlinear IPMs can also be designed to provide extremely high $\chi^{\left(3\right)}$ values of Kerr nonlinearity ( $>10^{-13}\;{\text{m}}^{2}/{\text{V}}^{2}$ ) and have response times below 2 ps [46], [47], [48].

**Figure 6:**
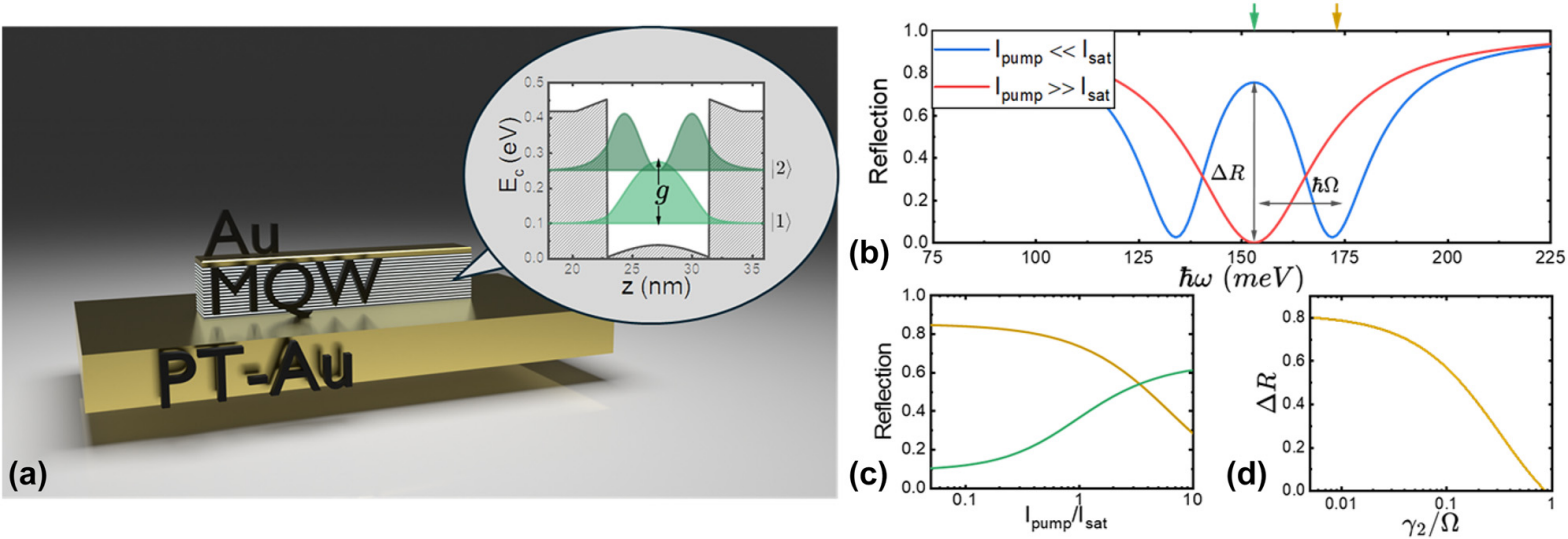
IPMs for optical power limiting and saturable absorption. (a) Schematic of the nonlinear metasurface for saturable absorption and power limiting reported in Ref. [47]. An incoming field (*s*_+_) feeds the nanoantenna, which in turn couples to the ISB transitions of the MWQ stack with a Rabi rate *g*, and determines the outgoing field (*s*_−_). (b) In the strong coupling regime, the low-intensity reflection spectrum of the system (blue line) displays the characteristic polaritonic splitting, leading to large reflection at *ω* = *ω*_*a*_. For high impinging intensities (red line), the ISB transitions in all quantum wells are fully saturated (*I*_pump_ ≫ *I*_sat_), and the reflection spectrum approaches one of the bare nanoantenna, featuring very low reflection at *ω* = *ω*_*a*_. The simulation uses the MQW parameters of the MQW shown in the inset in (a) with *ℏω*_*a*_ = 153 meV. (c) Reflection spectra of the metasurface versus the saturation level of the intersubband transition in the MQW for two different impinging frequencies: *ω* = *ω*_*a*_ (orange line in panel (c), also marked in panel (b) with the orange arrow) and *ω* = *ω*_*a*_ + Ω (green line in panel (c), also marked in panel (b) with the green arrow). (d) Maximal reflection contrast $\Delta R=R_{{\text{I}}_{\text{pump}}\ll I_{\text{sat}}}\left(\omega_{a}\right)-R_{I_{\text{pump}}\gg I_{\text{sat}}}\left(\omega_{a}\right)$ is projected to decrease with rising transition linewidth *γ*_2_ highlighting the importance of optimizing MQW materials for that parameter.

The schematic and the operating principle of IPMs that display giant Kerr nonlinear response are shown in Figure 6. The metasurface consist of double-metal patch antennas filled with a MQW heterostructure as shown in Figure 6(a). The intersubband transition between the ground and the first excited electron states in the MQW heterostructure is designed to have the same transition frequency as the dipole resonance of the patch antenna. The patch-antenna resonance and the intersubband transition couple to produce an intersubband polariton and the reflection spectrum of the metasurface will have two absorption dips associated with the two branches of the intersubband polariton as shown schematically with a blue line in Figure 6(b). At higher laser intensities, the intersubband transition saturates, resulting in a transition of the metasurface absorption spectrum to a single absorption peak associated with the patch-antenna resonance, see the red line in Figure 6(b). Depending on the operating wavelength, such a change in reflection spectrum results in either an increase or a decrease of the metasurface reflectivity with increasing pumping intensity, as shown in Figure 6(c).

### 4.1 Modeling intensity dependent polaritons

The reflectivity changes result from intensity-dependent saturation, necessitating a model that links the nanoresonator modes with the density matrix of the intersubband system. For this, we consider a metasurface with a unit cell shown schematically in Figure 6(a), consisting of a stack of MQW layers interacting with a metal patch antenna. For the analysis, we can use a coupled-mode theory combined with a Maxwell–Bloch model modified to account for absorption from higher-energy electron subbands [46]:

(2) $$\frac{d}{dt}a=\left[i\omega_{a}-\gamma_{r}-\gamma_{a}-\gamma_{s}\left(w+1\right)\right]a+iNgq+\frac{\sqrt{2\gamma_{r}}}{\hbar \omega A}s_{+},$$

(3) $$\frac{d}{dt}q=\left(i\omega_{q}-\gamma_{2}\right)q-igwa,$$

(4) $$\frac{d}{dt}w=4Im\left(qa^{*}\right)+\gamma_{1}\left(w+1\right),$$

(5) $$s_{-}=s_{+}-\sqrt{2\gamma_{r}}\left(\hbar \omega A\right)a.$$

In this model, the patch antenna is cast as a low-loss optical resonator with complex amplitude *a*, normalized such that ${\left|a\right|}^{2}$ represents the number of stored photons. It has a resonant frequency *ω*_*a*_ = 2*πv*_*a*_ with radiative and nonradiative decay rates *γ*_*r*_ and *γ*_*a*_, respectively. The optical resonance can be directly excited by an incident plane wave with a time-dependent amplitude *s*_+_ normalized so that ${\left|s_{+}\right|}^{2}$ represents the incident power per unit cell area *A*, and a carrier frequency *ω*. The outgoing back-propagating field *s*_−_ (Eq. (5)) results from a superposition of the directly reflected incident field and the field emitted by the optical resonance. The MQW is described by *N* identical two-level quantum oscillators, each with transition frequency *ω*_*q*_ = 2*πν*_*q*_, total decoherence rate *γ*_2_ and relaxation rate *γ*_1_, and coupled to the optical resonance with coupling rate $g=\frac{d_{12}}{\hbar }\sqrt{\frac{\hbar \omega }{2\varepsilon_{0}\varepsilon_{r}V}}$ , where *ε*_*r*_ is the relative permittivity of the MQW system at zero doping, *d*_12_ is the transition dipole moment from state 1 to state 2, and *V* is the mode volume. This effective two-level system describes the physical system depicted in Figure 6(a) in which a MQW heterostructure with *N* electrons is placed in a nanocavity of volume *V*. The state of the two-level system is described by the off-diagonal element of the reduced density matrix, denoted *q*, which describes the polarization of the transition, and by its inversion *w* = *n*_*e*_ − *n*_*g*_, where *n*_*g*_ and *n*_*e*_ are the electron populations in the ground and first excited electron levels in the MQW system, normalized to the total electron population. The term *γ*_1_ in Eq. (4) represents electron relaxation rate from excited to ground state in the MQW system, *γ*_1_ = 1/*T*_1_, where *T*_1_ is the excited state lifetime. Additionally, Eq. (2) contains an effective loss term $\gamma_{s}\left(w+1\right)$ [46], which accounts for the light absorption due to transitions of the electrons from the first excited state to higher electron states.

The intensity dependence of reflectivity is hereby driven by the value of *w*, approaching the value of −1 at low pumping intensities and producing a strongly coupled degenerate system (*ω*_0_ = *ω*_*a*_ = *ω*_*q*_) with a reflection spectrum $R\left(\omega \right)\equiv {\left|s_{-}\left(\omega \right)\right|}^{2}/{\left|s_{+}\left(\omega \right)\right|}^{2}$ that displays the characteristic Rabi splitting of upper and lower polaritonic modes as shown with the blue line in Figure 6(b). The Rabi splitting Ω occurs symmetrically around the central frequency *ω*_0_ as shown in Figure 6(b), where $\Omega \equiv \sqrt{N}g$ . For higher intensities, the value of *w* approaches 0 and the saturated ISB transitions are now decoupled from the optical resonance leading to a single minimum within the reflection pattern shown with the red line in Figure 6(b).

The maximal achievable change in reflectivity Δ*R* in these metasurfaces is fundamentally limited by different decay channels of the system *γ*_2_, *γ*_*a*_, *γ*_*s*_, and *γ*_*r*_. An ideal power limiter requires the condition *R* = 0, e.g., critical coupling for the metasurface antennas, in the high light intensity limit. This means that *γ*_*r*_ ≈ *γ*_*a*_ + *γ*_*s*_ at input frequency *ω* = *ω*_a_. In this case, it can be shown with the equations (2)–(5) that the maximal reflectivity contrast $\Delta R=R\left(w=-1\right)-R\left(w=0\right)$ at this frequency is given as [46], [47]:

$$\left|\Delta R\right|=\frac{{\left(1-\frac{\gamma_{s}\gamma_{2}}{\Omega^{2}}\right)}^{2}}{{\left(1+2\frac{\gamma_{a}\gamma_{2}}{\Omega^{2}}+\frac{\gamma_{s}\gamma_{2}}{\Omega^{2}}\right)}^{2}}\leq \frac{1}{{\left(1+2\frac{\gamma_{a}\gamma_{2}}{\Omega^{2}}\right)}^{2}},$$

where the upper limit of inequality is achieved when *γ*_*s*_ = 0. We note that the ratio *γ*_2_/Ω appears in all of the damping terms in Eq. (5) and thus MQW systems with a small transition linewidth *γ*_2_ and a high vacuum Rabi frequency Ω maximize the value of Δ*R*. The value of *γ*_*s*_ should also be minimized as much as possible [47]. For instance, Figure 6(d) shows the calculated Δ*R* as a function of *γ*_2_/Ω and for a fixed value of *γ*_*s*_ = 0 and *γ*_*a*_/Ω = 0.1. By switching to lower loss all dielectric nanoresonators *γ*_*a*_ ≪Ω can be reduced significantly the maximal performance of such a system comes down to the ratio of *γ*_2_/Ω.

To estimate the maximum value of Ω, we consider that the maximum population difference between the ground and the first excited states in the MQW system occurs when Fermi energy equals the state energy difference. Taking *E*_*f*_ = *ℏω*_*q*_ one obtains the corresponding electron sheet density of $N_{\text{s},\max}=\frac{m_{e}^{*}\omega_{q}}{\pi \hbar }$ per MQW period. One can further estimate the thickness of a quantum well to produce intersubband transition between the ground and the first excited state at a given photon energy *ℏω*_*q*_ as $L_{QW}=\sqrt{\frac{3\pi^{2}\hbar }{2m_{e}^{*}\omega_{q}}}$ and thus deduce the maximum electron density in the MQW system that maximizes the Rabi frequency Ω. In this way, one can estimate the maximum value of Ω achievable with the IPMs shown in Figure 4 as [47]:

(6) $$\Omega_{\max}=\omega_{q}\sqrt{\frac{e^{2}f_{12}}{2\pi^{2}\hbar \varepsilon_{0}\varepsilon_{r}}\sqrt{\frac{2m_{e}^{*}}{3\hbar \omega_{q}}}},$$

where $f=2m_{e}^{*}\omega_{q}d_{12}^{2}/\hbar$ is the oscillator strength, *ɛ*_*r*_ the relative permittivity, and $m_{e}^{*}$ the effective mass of the electrons in the well material in the MQW.

The value of Ω_max_ shows a weak dependence on the effective electron mass; thus, the choice of the well material in the MQW system is not of particular importance [49]. The relative dielectric constant can be assumed to be *ɛ*_*r*_ ≈ 10 for most suitable ISB systems [49]. Assuming *f*_12_ = 0.96, $m_{e}^{*}\approx 0.04m_{e}$ , where *m*_*e*_ is the mass of a free electron, and *ℏω*_*q*_ ≈ 150 meV, we obtain Ω_max_ ≈ 0.26*ω*_*q*_.

In the systems reported in Refs. [46], [47], a Rabi frequency of Ω = 0.13*ω*_*q*_ was used, which is about half the maximum achievable value (Ω_max_). This discrepancy is due to several factors. First, the doping level was approximately two times lower than needed for the optimal difference between *N*_1_ and *N*_2_. Second, the barriers in the MQW structure were roughly equal in thickness to the quantum wells (i.e., *L* ≈ 2*L*_*QW*_). While the first issue can be improved by using higher doping, and the width of the barriers could be minimized, these improvements would only lead to a modest increase in Ω. Furthermore, significantly reducing the barrier thickness is not viable because thinner barriers cause energy level anticrossing between adjacent quantum wells, increasing the intersubband transition linewidth. Given these considerations, significantly increasing the Rabi frequency Ω is not feasible without compromising other system aspects.

The best reflection contrast in the Kerr IPMs is thus produced by reducing the damping constants *γ*_2_, and *γ*_*s*_, and by minimizing the parameter *γ*_*a*_ linked to optical loss in the bare cavity, arising from ohmic losses in the metal and scattering at the nanoresonator walls, through optimizing the fabrication process to reduce the nanoresonator sidewall roughness.

The value of *γ*_*s*_ can be reduced by limiting the impact of the ISB transitions from the excited state to higher energy states. The intersubband transition dephasing rate *γ*_2_ appears in all the factors in Eq. (6), making its reduction crucial for improving metasurface performance. Typically, *γ*_2_ is over ten times higher than *γ*_1_, mainly due to roughness-induced scattering at quantum well/barrier interfaces, as well as electron–electron and phonon scattering.

### 4.2 High-contrast IPM-based saturable absorbers and power limiters

**Figure 7:**
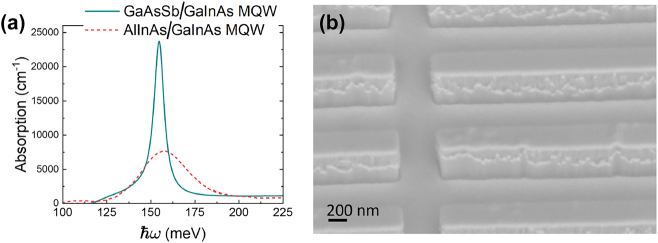
Improved IPMs for optical power limiting and saturable absorption reported in Ref. [47]. (a) Absorption spectra of intersubband transitions in MQWs based the Ga_0.53_In_0.47_As/GaAs_0.51_Sb_0.49_ heterostructure [47] in comparison to the MQW based on the Ga_0.53_In_0.47_As/Al_0.48_In_0.52_As heterostructure in Ref. [46]. (b) A scanning electron microscope image of the IMP reported in Ref. [47].

Reference [47] reported Ga_0.53_In_0.47_As/GaAs_0.51_Sb_0.49_ heterostructures with doping levels comparable to those in the earlier IPM systems based on Ga_0.53_In_0.47_As/Al_0.48_In_0.52_As heterostructures. The impact of this improvement is evident in the absorption spectral shown in Figure 7(a). The new materials system is expected to have a profound effect on a wide range of nonlinear IMPs. Leveraging on the extremely narrow linewidth of the intersubband transition, Ref. [47] reported saturable absorbers and optical power limiters based on IPMs with record-high reflection contrast system was designed around a vertical ISB transition within a single quantum well.

The MQW stack consists of 15 repetitions, each with an 8.2 nm-wide Ga_0.53_In_0.47_As well, separated by a 15 nm-wide GaAs_0.51_Sb_0.49_. Charge carriers in each well were introduced through two delta-doping regions, with a concentration of *n* = 6.5 ⋅ 10^11^ cm^−2^, located 2.3 nm from either side of the well. To reduce band bending effects near the metal contacts, Ga_0.53_In_0.47_As buffer layers with *n*-doping of 1 ⋅ 10^19^ cm^−3^ were added on both sides of the MQW stack. The bottom buffer layer is 5 nm thick, while the top layer, also serving as an etch stop during fabrication, measures approximately 15 nm. This design closely aligns with the earlier Ga_0.53_In_0.47_As/Al_0.48_In_0.52_As-based IMP for optical power limiting and saturable absorption ported in Ref. [46].

**Figure 8:**
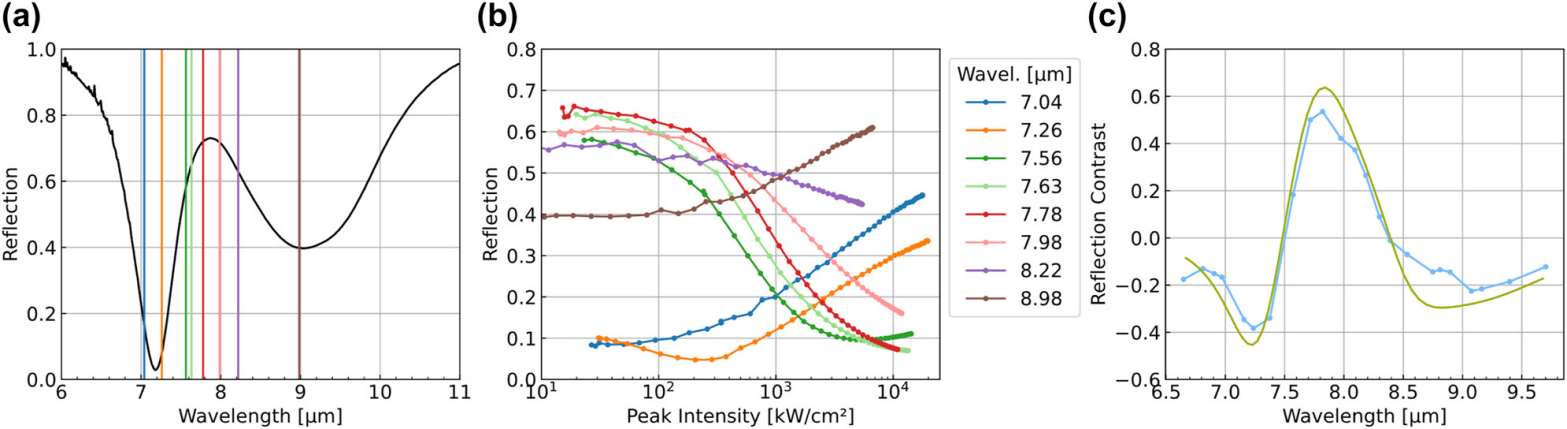
Experimental measurements of improved IMPs for optical power limiting and saturable absorption reported in Ref. [47]. (a) Measured low-power reflection spectra of the IMP. Colored lines indicate the wavelengths used in experimental testing. (b) Measured reflection versus impinging peak intensities at different selected frequencies. The excitation frequency in each measurement is color-coded (see color bar) and indicated by a corresponding vertical line in panel a. (c) Experimentally measured (blue dots) and numerically calculated (green line) reflection contrast versus impinging light wavelength.

The MQW system was processed into IPMs with a unit cell shown schematically in Figure 6(a). Figure 7(b) presents a scanning electron microscopy image of a fabricated sample. Figure 8(a) shows the measured reflection spectrum of the metasurface for the case when the resonant frequencies of the patch antenna and the intersubband transition are approximately equal (*v*_*q*_ = *v*_*a*_ ≈ 38 THz). The spectrum reveals two distinct reflection dips associated with the two branches of an intersubband polariton. Measurements of the IPM reflectivity as a function of impinging intensity at different wavelengths are shown in Figure 8(b). A tunable laser system with 2 ps laser pulses at 80 MHz repetition frequency was used for the measurements. Measurements for six different laser wavelengths corresponding to the color-coded vertical lines in Figure 6(a) are shown in Figure 6(b).

As expected (cf. Figure 6(c)), when the excitation frequency is near the two polaritonic reflectivity dips (*ν* = 38.36 THz, dark green curve), the IPM reflection decreases significantly with increasing intensity – from about 0.64 at low intensity (∼100 kW/cm^2^) to below 0.1 at ∼10 MW/cm^2^, achieving a reflection contrast of Δ*R* ≈ 0.54. For frequencies closer to the reflectivity dips, the reflection increases with intensity. For instance, at *ν* = 40.67 THz (dark orange), it rises from 0.1 to nearly 0.5.

At the lowest intensity (∼50 kW/cm^2^), non-negligible saturation of the ISB transition is evident, with some reflection curves already showing a nonzero slope. Hence, the achievable reflection contrasts may exceed those observed in Figure 8(b). Figure 8(c) shows the experimentally measured dependence of the reflection contrast Δ*R* between low and high-intensity illumination for a wide range of optical frequencies in comparison with the prediction of the model described by Eqs. (2)–(5) [47].

By adding geometrical asymmetries to these nonlinear limiting responses, it is also possible to break reciprocity in free-space propagation [50], realizing interesting avenues for bias-free, magnet-less free-space nonreciprocal routers [51].

## 5 Conclusion and outlook

The field of nonlinear intersubband polaritonic metasurfaces is now about 10 years old. Inspired by the brilliant ideas of Prof. Capasso in two independent fields of science and technology, quantum engineering of electronic transitions and photonic engineering of optical wavefronts, these nonlinear metasurfaces are now ready for prime-time. They offer giant and ultrafast nonlinearities, tunable by design, and accessible with ultralow power levels, without constraints stemming from phase matching. As such, they open totally new opportunities for the field of nonlinear optics. The opportunities demonstrated so far have been focusing on mid-infrared frequencies, which are the wavelengths naturally associated with intersubband electronic transitions – offering an exciting platform for the generation, manipulation, and mixing of infrared signals, key for applications in sensing, communications, THz generation, and thermal imaging. Yet, these concepts may be translated to higher frequencies by engineering the electronic response of the material and by looking into interband transitions. As another relevant quest, so far most of the examples of IPMs available in the literature, including those highlighted in this review, rely on reflective mode of operation, since they exploit a reflective ground plane to engage vertically polarized resonant fields. Yet, transmissive metasurfaces may be very appealing for applications aiming at imaging or to enable nonlinearity-induced noreciprocal responses [50]. All-dielectric designs that may be more amenable for transmissive operation may also lead to reduced insertion losses and enhanced efficiency for these devices, especially for operation at higher wavelengths. Overall, the field of nonlinear polaritonic metasurfaces is vibrant and highly relevant both from its highly interdisciplinary basic science standpoint and in terms of the opportunities for new technology. This rapidly expanding field of research owes its origin and impact to the visionary work of Prof. Capasso in QCL and in metasurfaces.
